# The roles of alpha oscillation in working memory retention

**DOI:** 10.1002/brb3.1263

**Published:** 2019-03-19

**Authors:** Elvis Wianda, Bernhard Ross

**Affiliations:** ^1^ Rotman Research Institute, Baycrest Centre Toronto Ontario Canada; ^2^ Department of Medical Biophysics University of Toronto Toronto Ontario Canada

**Keywords:** brain oscillations, cross‐frequency coupling, event‐related synchronization, magnetoencephalography, phase statistics, phase‐amplitude coupling, working memory

## Abstract

**Introduction:**

Brain processes of working memory involve oscillatory activities at multiple frequencies in local and long‐range neural networks. The current study addressed the specific roles of alpha oscillations during memory encoding and retention, supporting the hypothesis that multiple functional mechanisms of alpha oscillations exist in parallel.

**Method:**

We recorded magnetoencephalography (MEG) in 25 healthy young adults, who performed a variant of a Sternberg working memory task. A sequential list of five consonant letters was visually presented and was followed after a 2.0 s retention interval by a probe of a pair of two letters from the study list. Participants responded whether the probe pair was in same or reversed order in the list.

**Result:**

Reaction time (RT) was shortest for the first letters in the list, increased with increasing serial position, and shorter for the last position. RT was substantially longer for the probe in reversed order. Time‐frequency analysis of the MEG revealed event‐related desynchronization (ERD) of alpha oscillations during the encoding interval and an alpha power increase (ERS) during memory retention. Alpha ERD during encoding occurred at 10 Hz and ERS during retention at 12 Hz, suggesting different alpha mechanisms. Analysis of alpha coherence and alpha‐gamma cross‐spectral coupling, applied to MEG beamformer source activity, revealed connectivity across brain areas. Additionally, alpha‐gamma coupling identified centers of local computation. The connectivity between occipital and frontotemporal areas was correlated with alpha ERS during memory retention. Cross‐frequency coupling between alpha phase and gamma amplitude depicted a hierarchy of information flow from frontal to temporal and occipital brain areas.

**Conclusion:**

Alpha decrease during encoding indicates an active state of visual processing, while subsequent ERS indicates inhibition of further visual input for protecting the memory, and phasic timing of temporal and occipital gamma oscillations is related to a long‐range working memory networks.

## 1 introduction

Working memory (WM), defined as the ability to maintain and manipulate information in memory over a short period of time, is essential for a wide range of cognitive function such as language, learning, and general intelligence (Baddeley, 2012). Therefore, understanding the neural mechanisms underlying WM is of great interest. A promising concept of the neural mechanism of WM is that the operational stages of encoding, retention, and retrieval are associated with neural oscillations in various frequency bands. Previous studies supported the functional relevance of oscillations by showing that task demand modulated the magnitude of neural oscillations (Klimesch, 1996). Temporal and spatial properties of such modulations have been studied using event‐related modulation of the signal power in EEG or MEG, which are termed event‐related desynchronization (ERD) in the case of a power decrease and event‐related synchronization (ERS) in the case of an increase (Babiloni et al., 2005; Pfurtscheller & Lopes Da Silva, 1999).

Several studies showed ERD of alpha oscillations (8–14 Hz) related to memory function (Bonnefond & Jensen, 2012; Gevins, Smith, Smith, McEvoy, & Yu, 1997; Hanslmayr, Spitzer, Spitzer, & Bäuml, 2009; Klimesch et al., 1996; Krause, Lang, Lang, Laine, Kuusisto, & Pörn, 1996; Weiss & Rappelsberger, 2000). The decrease in alpha power indicates a state of desynchronization in which local neural assemblies become increasingly independent in preparation for a subsequent active process (Pfurtscheller, 1992). Following this interpretation, the reverse effect of alpha ERS has been suggested as reflecting a state of cortical inactivation (Pfurtscheller, Stancák Jr, Stancák, & Neuper, 1996). However, findings about the direction of alpha power change were not consistent across experimental studies, and the role of alpha oscillations during WM needs further clarifications.

Alpha power decreased during encoding in a visual WM task and the magnitude of ERD was correlated with memory load (Fukuda, Mance, Mance, & Vogel, 2015). However, a load‐dependent alpha increase was reported during the retention interval of WM (Jensen, Gelfand, Gelfand, Kounios, & Lisman, 2002). The latter two studies show that it is important to consider distinct effects on alpha oscillations during the different functional intervals of a WM task. The first report of alpha ERS during memory retention came from a WM study in which two different memory sets where used, that either remained consistent across trials, thus involving long‐term memory, or varied between trials and relied on a short‐term memory (Klimesch, Doppelmayr, Doppelmayr, Schwaiger, Auinger, & Winkler, 1999). ERS in the upper alpha band was observed only in the latter condition, which maximized short‐term memory demands. The authors interpreted the alpha ERS as indicating inhibition of a potential interference from the previous trials in the variable memory set condition. Further evidence supporting this explanation came from a study of a visually cued motor task, in which participants had to perform a finger movement or inhibit such response depending on the cue (Hummel, Andres, Andres, Altenmüller, Dichgans, & Gerloff, 2002). EEG recording in their study showed alpha ERS over the sensorimotor areas during the inhibition of the response and ERD during the actual response. Those results suggested that the increased alpha activity reflects inhibition of retrieving the stored motor memory traces in the somatosensory cortex, which is consistent with the concept that alpha ERS helps to block the retrieval of information from the previously stored trials. Thus, a current interpretation of alpha ERS is that alpha oscillations protect the new memory by inhibiting further sensory processing that could interfere with the stored information (Bonnefond & Jensen, 2012). As an alternative to the idling hypothesis of alpha, the authors suggested that the alpha increase plays an active functional role in preventing the flow of distracting information into areas which retain the memory items (Mazaheri et al., 2014). For instance, the inhibition or disengagement of occipital‐parietal areas could serve to suppress input from the visual stream, which would otherwise, disturb the maintenance of WM in frontal areas. Consistent with studies in the visual modality, an auditory study predicted right hemispheric dominance for processing memory of pitch, and the authors interpreted a left‐lateralized increase in 5–12 Hz activity as functionally disengaging left temporal regions (Van Dijk, Nieuwenhuis, Nieuwenhuis, & Jensen, 2010).

Given the limited capacity of WM, protecting the memory from interferences seems crucial for the successful WM performance. However, recent studies, testing whether the increased alpha activity could serve such an inhibitory function did not support the inhibition hypothesis (Poch, Valdivia, Valdivia, Capilla, Hinojosa, & Campo, 2018; Schroeder, Ball, Ball, & Busch, 2018). Thus, current research focuses more on the role of alpha oscillations in controlling the timing within neural networks (Klimesch, 2012). Findings of oscillatory activity in the gamma frequency range (30–120 Hz) during WM maintenance (Howard et al., 2003) suggested that a coordinated interplay between alpha and gamma oscillations supports WM function (Roux & Uhlhaas, 2014). The role of gamma oscillations for cognition and memory has been established more thoroughly than for alpha. For example, gamma band activity was observed during the delay interval in a delayed matching‐to‐sample task and was absent in the control task (Tallon‐Baudry, Bertrand, Bertrand, Peronnet, & Pernier, 1998). This finding supported the hypothesis that visual objects are represented by distributed cell assemblies, synchronized in their gamma band activity (Tallon‐Baudry, Bertrand, Bertrand, Delpuech, & Pernier, 1997). Similarly, load‐dependent gamma band activity was found in a visuospatial WM task, in which participants were required to memorize the positions of red disks only and to ignore the positions of the blue disks (Roux & Uhlhaas, 2014). A consistent relationship between the amplitude of gamma oscillation and the number of target items suggested that gamma oscillations are implicated in the maintenance of relevant WM information (Daume, Gruber, Gruber, Engel, & Friese, 2017). This role of gamma oscillation seems universal across sensory modalities because a gamma increase had been reported for secondary somatosensory areas during retention in a somatosensory WM task (Haegens, Osipova, Osipova, Oostenveld, & Jensen, 2010). In a study of auditory pattern memory, induced gamma activity was enhanced over left inferior‐frontal and anterior‐temporal regions during retention, while this was not the case in a control condition (Kaiser, Ripper, Ripper, Birbaumer, & Lutzenberger, 2003). The authors interpreted their findings that gamma activity is a correlate of cortical networks involved in the mental representation of sensory information. Recent findings that slow alpha waves and fast gamma activity occurred simultaneously during WM maintenance were discussed as an interaction of alpha and gamma for serving as a mechanism of neural communication (Canolty & Knight, 2010). It was thought that alpha waves modulate the excitability of neural networks that produce high‐frequency oscillations. In this mechanism of cross‐frequency coupling, alpha oscillations seem to play the leading role in controlling gamma.

The aim of the current study was providing further support for emerging concepts about the roles of oscillatory activity underlying WM. We hypothesized that multiple functional roles of alpha could be observed simultaneously from the same experiment. Differences in the experimental paradigms in previous studies may have contributed to differences in the observed alpha effects. We implemented a modified Sternberg paradigm (Sternberg, 1966) because the encoding, retention, and retrieval intervals are well separated compared to other WM experiments. This allowed for separate analyses of oscillatory MEG activities for the subsequent WM intervals. First, we analyzed the temporal dynamics of alpha activity during the different stages of WM. Specifically, we expected alpha during the retention interval being involved in the timing of neural activity. Therefore, time intervals of increased alpha activity would be more phasic compared to intervals of decreased alpha activity. We identified underlying cortical sources with beamformer source analysis, measured alpha coherences, and alpha‐gamma coupling between the cortical sources, and compared those connectivity measures with alpha ERS and ERD.

## 2 materials and methods

### 2.1 Participants

Twenty‐five adults (10 female, 15 male) between 21 and 43 years of age were recruited for the study. Participants reported good health, no history of neurological or psychiatric disorders, and did not require correction for normal vision. They provided written consent after receiving a full explanation of the study, which was approved by the Research Ethics Board at Baycrest Centre.

### 2.2 Experimental task

**Figure 1:**
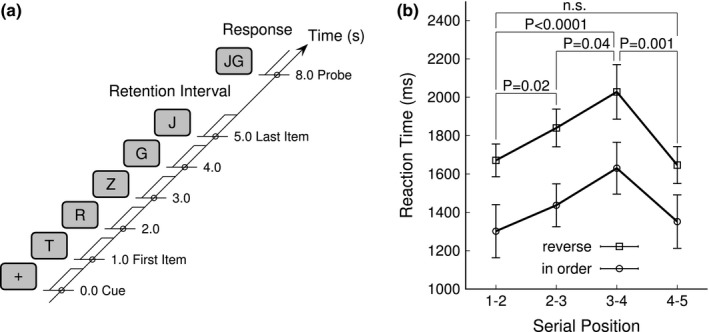
Working memory experiment. (a) Time course of the experimental paradigm. After a start cue (+), the five letter list items were presented sequentially for 700 ms duration and 1,000 ms inter‐onset interval. The retention interval between the offset of the last list item and the probe onset lasted for 2,300 ms. The cue onset served as zero‐time for the data analysis. (b) Group mean reaction time in relation to the serial position and order of the probe pair. The error bars denote the 95% confidence intervals of the group mean

The sequence of visual stimuli for the modified Sternberg paradigm started with a cue symbol (+), followed by a sequential list of five capital letters, a blank screen retention interval, and a probe. The study list was a unique combination of randomly chosen consonant letters. Vowels were excluded to make it less likely that participants chunked the list into a word. The probe was a pair of two letters, which had been presented next to each other in the study list. Participants had to decide whether the probe items had been presented in same or reversed order in the study list. Cue and list items were presented for the duration of 700 ms with an inter‐onset interval of 1,000 ms, which resulted in a total duration of 5,700 ms of visual stimulation (Figure 1a). Participants held the studied list in memory during the retention interval of 2,300 ms between the offset of the last list item and probe onset. The retention interval was chosen to be longer than 1,500 ms to reduce the effect of the most recent list item being easily remembered (Olton & Samuelson, 1976). The probe was presented for 1,000 ms, and participants responded with right‐hand button press within 2,000 ms after the probe onset. A shorter reaction time was expected when the letters of the probe pair were in same order as in the list than for the more difficult task of finding the reversely ordered probe pair. No feedback was given whether the response was correct or not. The next sequence was initiated 4,000 ms after the button press. Thirty WM sequences were performed within an experimental block of 7.5 min duration. Each participant completed six blocks within a session. Stimulus presentation was controlled by Presentation software (Neurobehavioural Systems, Inc., Berkeley, CA). The stimuli were projected onto a back‐projection screen of 50 cm in diagonal at distance of 60 cm in front of the participant. The letters were black on a gray background and had a height of 80 mm, corresponding to a visual angle of 7.6 degrees. For precise timing of the stimulus events, picture onsets were detected with a photodiode.

### 2.3 MEG recording

Magnetoencephalography was recorded in a quiet magnetically shielded room using a 151‐channel whole‐head axial gradiometer‐type MEG system (CTF‐MEG, Port Coquitlam, BC, Canada) at the Rotman Research Institute. Two MEG channels were disabled for technical reasons. Participants were comfortably seated in an upright position with the head resting inside the helmet‐shaped MEG device. The magnetic field data were low‐pass filtered at 200 Hz, sampled at 625 Hz, and stored continuously. The participant's head position relative to the MEG sensor was registered at the beginning and end of each recording block using three small electromagnetic coils, attached to fiducial points at the naison and left and right pre‐auricular points. For an experimental block in which the fiducial positions were different in any direction by more than 5 mm from the mean, large head movements were assumed, and the block was repeated. The mean of both fiducial positions also defined the head‐based coordinate system with the origin at the midpoint between the bilateral pre‐auricular points. The posterior‐anterior x‐axis run from the origin to the nasion, the y‐axis run from right to the left ear, perpendicular to x in the plane of the three fiducials, and the inferior‐superior z‐axis run perpendicular to the x‐y plane toward the vertex. Trigger signals, indicating time points and types of stimulus events were recorded simultaneously with the MEG.

### 2.4 Data analysis

The data analysis was aimed at showing how changes in the magnitude of alpha oscillation relate to the different stages of WM processes, how these alpha power changes manifest across the brain, whether alpha coherence between sensors would indicate functional connectivity, and whether temporal coupling between alpha and gamma rhythms would indicate a role of alpha for precise neural timing. The MEG data were preprocessed to remove eyeblink and heartbeat artifacts. First, the time points of the artifacts were identified using the independent component analysis function **fastica** from the EEGLAB toolbox (Delorme & Makeig, 2004). Spatiotemporal templates were constructed as the first principle components of averaged artifacts and were used to eliminate artifacts in the continuous data (Kobayashi & Kuriki, 1999). The preprocessed MEG data were then parsed into epochs of 16 s duration, equivalent to 10,000 samples. The cue onset defined the zero‐time. Each epoch contained 2.0 s of pre‐stimulus time, the encoding interval of 5.7 s of visual stimulation, the 2.3 s retention interval, and a 6.0 s interval, consisting probe presentation, memory recall, decision‐making, response, and post‐response times.

### 2.5 Time‐frequency analysis

Time‐frequency analysis was applied to all epochs of the MEG sensor data to study the temporal dynamics of oscillatory brain activity and its spatial variation across the sensor domain. The time‐frequency representation was calculated at 64 frequencies, logarithmically spaced between 2 Hz and 60 Hz, using a complex Morlet wavelet (Kronland‐Martinet, Morlet, Morlet, & Grossmann, 1987; Samar, Bopardikar, Bopardikar, Rao, & Swartz, 1999). The full width of the wavelet at half of its maximum was equivalent to two cycles at 2 Hz and six cycles at 60 Hz. This approach of varying the wavelet width across frequencies was suitable to account for the trade‐off between time and frequency resolution across the frequency range of interest (Bruns, 2004). The 10‐Hz wavelet had a half‐intensity width of 389 ms corresponding to the bandwidth of 2.57 Hz. Wavelet coefficients *w(t,f) *were calculated at each 8th sample in time by convolving the time series with the wavelet. Before applying the wavelet transform to each trial of an experimental block, the averaged signal was subtracted to reduce the effect of evoked responses on the power measures. The resulting 1,250 × 64 time‐frequency coefficients for each trial, sensor, and participant were stored for further analyses. All data analyses were performed with in‐house developed matlab functions.

### 2.6 Time courses and topographic maps of ERD/ERS

ERD and ERS were computed as signal power changes relative to the signal power in the baseline interval for each time‐frequency bin and each MEG sensor (Graimann & Pfurtscheller, 2006). The baseline was the 2‐s interval preceding the onset of the visual cue. Signal power *P(t,f)* was calculated as the product of each wavelet coefficient and its conjugate complex. For each frequency bin, the signal power was normalized relative to the mean power $P_{B}$ in the baseline interval and expressed in percent: $ERS=100\times \left(P\left(t,f\right)/P_{B}\left(f\right)-1\right)$. Negative values were termed as ERD. ERD/ERS values were averaged across trials, repeated blocks, and participants. Alternatively, ERD/ERS is sometimes expressed as the logarithm of the signal power ratio and scaled in decibels (Makeig, 1993). The percent and the logarithmic measures are closely similar for small changes, for example, ±10%, because of the approximation, ln(x-1)≈x,∈x≪1. For larger signal power changes, the logarithmic measure numerically emphasizes on ERD whereas percent changes numerically emphasize ERS.

We analyzed alpha ERD/ERS both in the sensor domain and after applying a MEG beamformer analysis in the source domain. The source domain analysis allowed for separating the activity in multiple cortical sources for studying connectivity properties. We employed the sensor domain analysis for studying the global properties of alpha oscillations. First we aimed at providing a spatial map of consistent alpha ERD/ERS across the head. Therefore, we applied a principal component analysis (PCA) to the multivariate ERD/ERS data after averaging across all participants. The PCA decomposed the 149 time series of ERD/ERS in the 8 Hz to 14 Hz frequency band into principal components (PC) consisting of a single time series. The corresponding topographic map of factor loads indicated how strongly the temporal pattern of ERD/ERS was represented at each sensor. A correlation between the individual time series and each PC, measured the similarity between individual ERD/ERS and the group mean PC. We applied a *t* test to the similarity index for testing whether the individual participants contributed consistently to the group mean ERD/ERS map. We corrected the p‐values for the false discovery rate using the **mafdr** Matlab function.

### 2.7 Distinct frequency bands for alpha ERD and ERS

We measured the peak frequencies of alpha ERD and ERS separately for the encoding and retention intervals because of previous reports that the upper and lower alpha frequencies are differently related to the memory process (Klimesch, Doppelmayr, Doppelmayr, & Hanslmayr, 2006; Petsche, Kaplan, Kaplan, Stein, & Filz, 1997). Also, different peak frequencies for ERD and ERS could indicate that different alpha processes are involved in ERD and ERS. One caveat for interpretation of ERD/ERS is that the numerical values of percent changes may be large in case of low signal power in the baseline interval while absolute power changes are small. Therefore, we analyzed absolute spectral power changes and compared those to the center frequencies of ERD and ERS effects. We measured the individual alpha frequencies as the center of gravity in the 7 Hz to 14 Hz alpha band in the averaged signal power in six regions of interest of frontal, central, and occipital sensors above the left and right hemispheres. The alpha frequencies were calculated for the signal power in the baseline interval (–2.0 s to 0), memory encoding (1.0 s to 6.0 s), and retention (6.0 s to 8.0 s) and for ERD/ERS in the encoding and baseline interval. A two‐way repeated measures ANOVA was applied to the signal power data with the factors “region of interest” (six levels) and “time interval” (three levels). The ANOVA for the ERD/ERS had only two levels for the factor “time interval.” We performed post hoc *t* tests and calculated confidence intervals for the ERD/ERS frequencies using bootstrap resampling.

### 2.8 Effect of memory load on alpha ERD/ERS

Previous research showed increased alpha ERS with increasing memory load (Gomarus, Althaus, Althaus, Wijers, & Minderaa, 2006). In our study, the memory load increased sequentially with the increasing numbers of letters in the study list. We analyzed the ERD/ERS peak amplitudes during the encoding interval to study the effect of the memory load. For the first four visually presented letters in the list, we measured the individual peak amplitudes of ERD/ERS in clusters of occipital sensors in the left and right hemispheres. We performed a three‐way ANOVA with the factors “hemisphere” (left, right), “response type” (peak, trough), and “letter position” (1st to 4th).

### 2.9 SAM source analysis

For studying the role of alpha oscillations for brain connectivity, we performed a whole brain source analysis. Source activity was reconstructed with synthetic aperture magnetometry (SAM) (Robinson & Vrba, 1999). SAM is based on a linearly constrained minimum variance beamformer (Van Veen, Van Drongelen, Van Drongelen, Yuchtman, & Suzuki, 1997). Participants' head shapes were obtained with a 3‐D digitization device (Polhemus Fastrak, Polhemus, Colchester, VT). Individual head models for the beamformer were constructed by locally approximating spheres for each MEG sensor to the digitized head shape. A validated procedure of using standard brain and individual head models (Steinstraeter et al., 2009) was used to co‐register the source images with a standard anatomical MR (colin27) (Holmes et al., 1998). The standard MRI was warped into the individual head shapes using the Brainstorm software (Tadel, Baillet, Baillet, Mosher, Pantazis, & Leahy, 2011). A set of weighting coefficients was determined for 72 regions of interest (ROIs) (Bezgin, Vakorin, Vakorin, Opstal, McIntosh, & Bakker, 2012; Kötter & Wanke, 2005). The linear combination of the weighting coefficients with the MEG data resulted in virtual sensor waveforms of the source activity at each ROI. We applied the same time‐frequency analysis that had been used in the sensor domain to the source waveforms.

### 2.10 Weighted phase‐lagging index (wPLI)

For testing the hypothesis that alpha oscillations are involved in functional connectivity between brain areas, the coherence of alpha oscillations between the brain source signals was calculated over the time course of the WM task. Alpha coherence was measured using the weighted phase‐lagging index (wPLI) (Vinck, Oostenveld, Oostenveld, Wingerden, Battaglia, & Pennartz, 2011) which is an extension of the phase‐lagging index (PLI) (Stam, Nolte, Nolte, & Daffertshofer, 2007). PLI measures the asymmetry of the distribution of phase differences between two signals and describes the consistency with which the phase of one signal is leading or lagging relative to the phase of the other signal. By weighing each phase difference according to the magnitude of the lag, phase differences around zero contribute minimally to the calculation of the wPLI. This procedure reduces the probability of detecting false positive connectivity in the case of volume conducted noise sources with near‐zero phase lag and increases the sensitivity in detecting phase synchronization (Vinck et al., 2011). The wPLI approach showed best performance in the presence of noise compared to other phase statistics (Wianda & Ross, 2016). The weighting factor is the magnitude of the imaginary cross‐spectrum. The complex cross‐spectrum *C(t,f)* between two sources with complex wavelet coefficients *X(t,f)* and *Y(t,f) *was computed as C(t,f)=X(t,f)∙Y(t,f)∗, where * indicates the complex conjugate. The wPLI was computed as.

$$wPLI=\frac{\left|E\left\{\left|imag\left(C\right)\right|sgn\left(imag\left(C\right)\right)\right\}\right|}{E\left\{\left|imag\left(C\right)\right|\right\}}$$

The wPLI was calculated for every pair of the 72 sources for the 12‐Hz time‐frequency coefficients and the 1,250 samples in time. This analysis resulted in a stack of 1,250 connectivity matrices with a dimension of 72 × 72 for each participant. For comparing connectivity during the retention interval, encoding, and pre‐stimulus baseline, wPLI values were averaged across the time intervals of 6.0 s to 8.0 s, 1.0 s to 6.0 s, and −2.0 s to 0, respectively, reducing the data to three 72 × 72 connectivity matrices for each participant.

For estimating the effect size of the connectivity measures, we compared the group mean wPLI for all elements of the connectivity matrix against a maximum obtained from randomized surrogate data. For each trial, we added a random phase in the range of ‐π to π to the data, calculated the wPLI as for the original data, and identified across all 72 × 72 source pairs the maximum of the group mean surrogate wPLI for the retention time interval. We estimated the confidence interval for the mean of the original wPLI by bootstrap randomization across participants and compared the lower 95% bound against the maximum in the surrogate data. The networks of the strongest connections were visualized using the BrainNet toolbox (Xia, Wang, Wang, & He, 2013).

For testing the overall change in connectivity between the three time intervals and differences between hemispheres, we obtained a univariate connectivity measure from a PCA applied to the connectivity matrix and correlation with individual connectivity matrices. Differences between the time intervals for the univariate measure were assessed with permutation tests (*n* = 1,000) across participants. A simple measure of connectedness was obtained for each ROI as the mean across the corresponding row of the connectivity matrix. For testing the hypothesis that connectedness depended on the level of ERS, we performed a linear regression of the ERD/ERS data on the connectedness for each ROI.

### 2.11 Alpha‐gamma phase‐amplitude coupling (PAC)

The relationship between the magnitude of gamma activities and the phase of alpha oscillation was analyzed with the cross‐frequency coupling method, which estimates the strength of pairwise interactions between two signals at different frequencies, both between and within sources (Buzsáki, 2010; Buzsáki, Logothetis, Logothetis, & Singer, 2013; Canolty & Knight, 2010). Cross‐frequency coupling between the phase of a low‐frequency signal and the amplitude of a higher frequency signal is termed PAC and has been applied most successfully (Cohen et al., 2009; Osipova, Hermes, Hermes, & Jensen, 2008; Voytek et al., 2010). Specifically, PAC tests whether the amplitude of gamma oscillation in a signal *y(t,f_γ_) *depends on the alpha phase in *x(t,f_α_)*. We employed a PAC algorithm in the time domain. The beamformer source signals were band‐pass filtered in the alpha and gamma frequency bands by convolution with FIR filters, designed with the **fir1** Matlab function. For exploring the properties of the cross‐frequency coupling, the frequency for phase (alpha) was varied between 5 Hz and 20 Hz in 1‐Hz steps, the frequency for the amplitude (gamma) was varied between 30 Hz and 150 Hz in 5‐Hz steps. While the alpha band‐pass filter was in the narrow range between 0.85 and 1.18 times the alpha frequency, the gamma band‐pass filter was defined by gamma frequency ±1.2 times the alpha frequency. Thus, the gamma bandpass included the upper and lower sidebands of the amplitude‐modulation spectrum (Aru et al., 2015). The Hilbert transform was applied to obtain complex signals. The time points of the peak maxima of the band‐pass filtered alpha signal were taken as phase references. The gamma signal was parsed into short epochs with the duration equal to four cycles of the alpha frequency. Each epoch was centered at the alpha maximum and was fitted with a complex wavelet at the alpha frequency by calculating the dot product between the gamma amplitude and wavelet. The outcome measure of this procedure was the phase of the gamma envelope relative to the alpha peak reference. We used circular statistics to reject the null hypothesis of a uniform phase distribution, indicating no phase relation between the gamma amplitude and the alpha phase. Mapping the outcome measure of the circular z‐score (Fisher, 1995, p 70) across the frequencies for phase and amplitude resulted in the comodulogram. Cross‐frequency comodulograms were calculated for all pairs of 72 × 72 sources for all individual participants. Comodulograms under the null hypothesis were obtained from surrogate data by adding trial by trial a random delay in the range between plus and minus a cycle of the alpha frequency to the gamma data. This technique removed the phase relation between the alpha and gamma data without disturbing the temporal dynamics of the spectral properties of the signal (Scheffer‐Teixeira & Tort, 2016).

Several authors cautioned that cross‐frequency measures are subject to interpretation and may result from nonlinearity or other events in the signals under consideration but not from neural interaction (Gerber, Sadeh, Sadeh, Ward, Knight, & Deouell, 2016; Hyafil, 2015; Kramer, Tort, Tort, & Kopell, 2008). Therefore, we calculated the bicoherence as an alternative method for cross‐frequency analysis and compared the different methods. The bicoherence was calculated from multiple Fourier transforms over 50% overlapping intervals of 500 ms duration across the WM maintenance interval between 6.0 and 8.0 s. The bispectrum was defined as $B\left(f_{1},f_{2}\right)=X\left(f_{1}\right)∙X\left(f_{2}\right)∙X^{*}\left(f_{2}+f_{1}\right)$ with the complex Fourier transform *X* and its conjugate X* (Sigl & Chamoun, 1994) and the bicoherence $BiCoh=\sum B\left(f_{1},f_{2}\right)/\sum \left|B\left(f_{1},f_{2}\right)\right|$ (Hayashi, Tsuda, Tsuda, Sawa, & Hagihira, 2007). Bicoherence was calculated for f_1_ = 5…20 Hz and f_2_ = 20…150 Hz.

For the group analysis, we considered two gamma frequency bands. First, we calculated PAC between 12‐Hz alpha and 45‐Hz gamma. Second, we averaged the individual comodulograms between 12 Hz and 14 Hz for alpha and between 60 Hz and 100 Hz for gamma.

### 2.12 Asymmetry of cross‐frequency coupling

Provided the hypothesis that the alpha phase controls the gamma amplitude (Fries, 2015), an asymmetry of PAC could be related to the directionality of neural communication. Specifically, we would interpret the asymmetry $PAC\left(x_{\propto },y_{\gamma }\right)>PAC\left(y_{\propto },x_{\gamma }\right)$ as alpha in area *x* is controlling gamma in area *y* more than vice versa. To test the asymmetry, we performed a student's *t* test between *PAC*(x,y) and *PAC*(y,x) and corrected p‐values for the false discovery rate using the Matlab function mafdr.

## 3 results

### 3.1 Behavioral performance

The group mean reaction time (RT) increased with increasing serial position of the probe in the study list, with the exception, that the RT for the last position was shorter again. RT was generally longer for the probe in reverse order (Figure 1b). A repeated measures ANOVA with the factors “probe order” (two levels) and “serial position” (four levels) revealed main effects of “probe order” (*F*(1,24) = 61.0, *p* < 0.0001) and of “serial position” (*F*(3,72) = 8.85, *p* < 0.0001) but no interaction between both factors (*F*(3,72) = 0.7, *p* = 0.6, n.s.). Pairwise comparisons showed significance for the RT increase when the serial position of the probe was shifted from (1–2) to (2–3) (*t*(23) = 2.47, *p* = 0.021) and from (2–3) to (3–4) (*t*(23) = 2.21, *p* = 0.037) and for the RT decrease between the positions (3–4) and (4–5) (*t*(23) = 3.60, *p* = 0.0014). RT for the first and last positions were not different (*t*(23) = 0.18, *p* = 0.9, n.s.). The RT was in mean 366 ms longer for the reversed probe (*t*(23 = 7.8, *p* < 0.0001). The behavioral data, showing the effect of serial position and the recency effect, indicate that the participants performed the WM task. The longer RT for the revered probe order suggests that participants performed an additional task of mentally manipulating the probe.

### 3.2 Time courses and topographic maps of ERD/ERS

**Figure 2:**
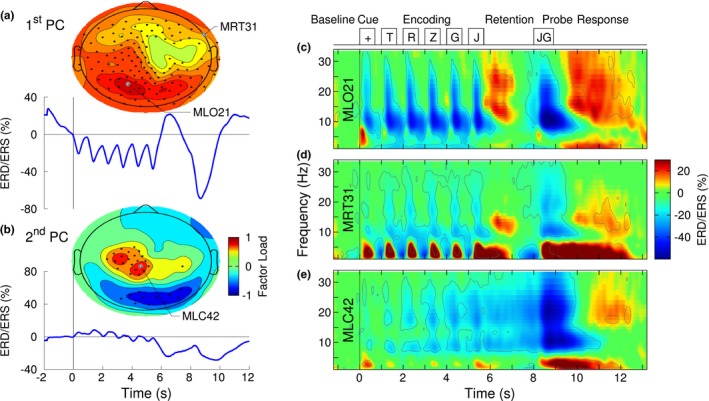
Overview of the ERD/ERS time‐frequency analysis. (a) Time course and topographical map of the first principal component (PC) of a principal component analysis (PCA) applied to the grand averaged ERD/ERS data. Filled circle symbols indicate the sensors at which the first PC was consistently represented in the individual data at *p* < 0.001. Occipital and parietal sensors contributed predominantly to the first PC. The maximum occurred at the the sensor MLO21. (b) The second PC was predominant at left central sensors above the sensorimotor cortex. Its time course was less modulated by the visual stimuli compared to the first PC. (c) Time‐frequency representation of grand mean ERD/ERS observed with the occipital sensor MLO21. The contour lines indicate the *p* = 0.001 level of a *t* test, applied across participants. The time course of visual stimuli is shown as reference on top. (d) Time‐frequency map of grand mean ERD/ERS at the frontotemporal sensor MRT31, and (e) the sensor MLC42 above left central areas

The PCA applied to grand averaged time series of ERD/ERS provided a global overview about how the temporal modulation of alpha power during the different stages of WM was represented in spatial patterns around the head. The time course of first PC, accounting for 86% of the variance, is shown in Figure 2a. During the encoding interval, alpha power decreased after the presentation of each visual stimulus, reaching the minimum in mean at 260 ms (95% CI = ±27.0 ms) after stimulus onset, which was immediately followed by a partial rebound with a maximum at 750 ms (95% CI = ±26.5 ms) latency. In contrast to the prominent ERD during the encoding interval, alpha ERS occurred during memory retention. Then again, alpha ERD was prominent during and after the probe presentation. The topographic map, corresponding to the first PC, depicted that ERD/ERS from occipital‐parietal areas, as well as from frontal areas contributed to the first PC. Circle symbols in Figure 2a indicate sensors, at which the first PC was consistently represented in the individual data at *p* < 0.001. The topographic map of the factor load of the second PC, accounting for 10% of the variance, showed major contributions from sensors above left central areas, corresponding to the sensorimotor cortices, contralateral to the responding right hand (Figure 2b). The time course of the second PC showed only minor variations during the memory encoding and retention intervals. However, a strong alpha ERD occurred before and after probe presentation, suggesting an involvement in response preparation and execution. Further PCs, accounting in total for less than 4% of the variance, were not consistently represented in the individual ERD/ERS data.

Visualization of the grand averaged time‐frequency map of ERD/ERS obtained from the left occipital sensor MLO21, which showed the largest factor load for the first PC, revealed six intervals of ERD at alpha frequencies, which followed the onsets of the visual cue and the five list items, as well as a strong ERD after probe presentation (Figure 2c). The initial phase of those ERD intervals showed a spectral spread into the beta range. In contrast to alpha ERD during encoding, the retention interval was characterized by ERS at alpha frequencies, also extending into the beta range. Informed by previous literature, one focus of data analysis was on the alpha ERS during memory retention. The time‐frequency map provided the first hint about a possibly higher center frequency for the ERS compared to ERD during memory encoding. Similar observations of alpha ERD during encoding and ERS during retention were made for the frontotemporal sensor MRT31 (Figure 2d). However, the magnitudes of ERD/ERS were generally smaller. Alpha ERD was concentrated in the time intervals immediately following the visual stimulus presentation. Moreover, the frontotemporal sensor showed distinct intervals of theta ERS following the visual stimuli, which was also strongly expressed after the presentation of the last list item and continued during memory retrieval after the probe occurred. A different time course of alpha ERD/ERS was observed from the sensor MLC42 which had the largest factor load for the second PC above left central areas. (Figure 2e). The modulation of ERD by the visual stimuli was less expressed in this sensor. Instead, ERD developed during the time interval of stimulus presentation, continued during the retention interval and was strongest after the probe presentation. The most noticeable difference to the occipital ERD/ERS map was the absence of ERS during the maintenance interval. Moreover, ERD extended into the beta range.

### 3.3 Alpha ERS during memory retention

**Figure 3:**
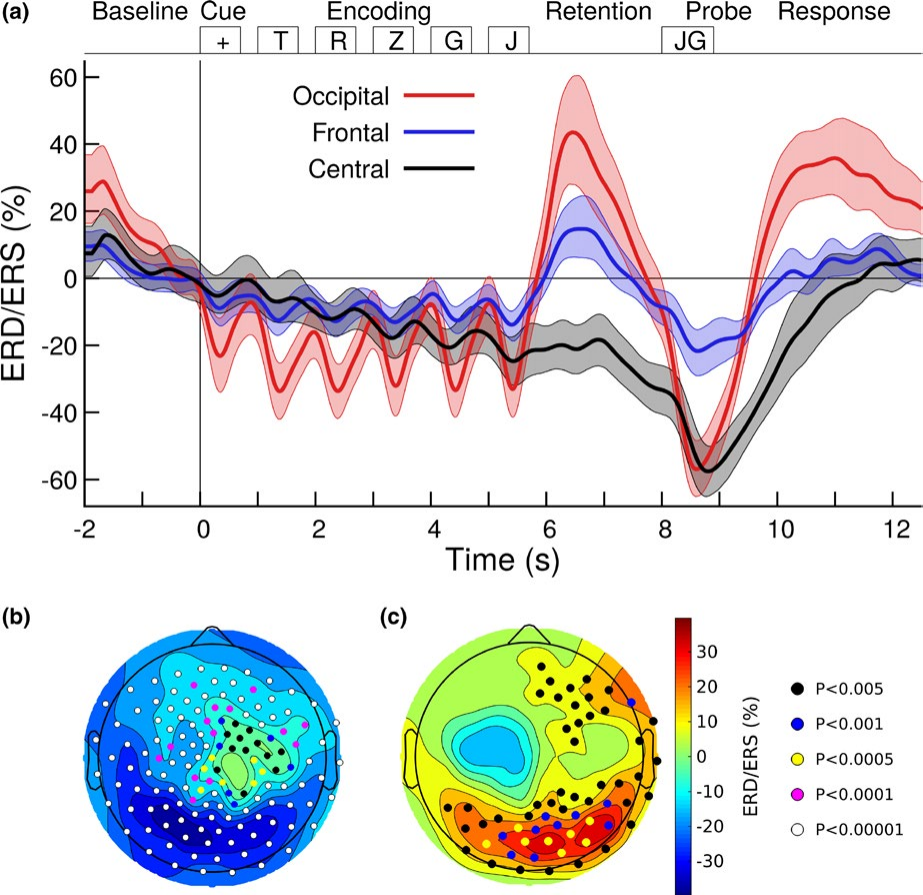
Alpha ERS during memory encoding and ERS during retention. (a) Group mean time series of ERD/ERS in the alpha band (8–12 Hz), obtained at the occipital sensor MLO21, the central sensor MRT31, and frontal MLC42. The shaded areas indicate the 95% confidence intervals for the group mean. (b) The topographic map of alpha ERD/ERS, averaged across the encoding interval (1.0 s–6.0 s), is dominated by cool colors, indicating ERD. Sensors, at which ERD reached the *p* < 0.005, are indicated with circle symbols. (c) Alpha ERD/ERS during the retention interval (6.0 s–8.0 s). Warm colors in occipital and frontal sensors indicate predominant alpha ERS. Sensors with ERS at *p* < 0.005 are indicated with circle symbols. Significant alpha power increase during the retention interval was observed at occipital and frontal sensors

For quantitative analysis of ERD/ERS in specific time intervals of the WM task, nonparametric bootstrap resampling with replacement was applied to the ERD/ERS data across the *n* = 25 participants. Figure 3a illustrates the time courses of alpha ERD/ERS in selected frontotemporal, central, and occipital sensors. Shaded areas indicate the 95% confidence intervals (CIs) for the group mean. The 95% CI for frontotemporal and occipital sensors did not include zero during the stimulus presentation and memory retention, indicating significant effects of alpha ERD and ERS. During memory retention, the CIs of frontotemporal and occipital sensors were nonoverlapping with each other, indicating significantly larger effect size occipital than in frontal sensors. In contrast, the left central senors showed no effect of ERS.

The *t* tests for alpha ERD during the encoding interval and ERS during memory retention revealed how consistent individuals contributed to ERD and ERS at the various MEG sensors. ERD during encoding was maximally expressed in left occipital sensors, however, was prevalent across the whole sensor array, except right central areas (Figure 3b). Alpha ERS during the retention interval was maximal in right occipital sensors. However, a cluster of sensors above right frontotemporal areas showed ERS effect sizes at *p* = 0.005 (Figure 3c).

### 3.4 Distinct frequency bands for alpha ERD and ERS

We examined whether ERD and ERS occurred at different center frequencies within the alpha band in various brain regions. For example, visual inspection of the time‐frequency map in Figure 2c revealed that the center frequency of ERS during memory retention was higher than the peak frequency of ERD during the encoding interval. Moreover, ERS during retention extended into the beta range, indicating the involvement of oscillations beyond the alpha band.

**Figure 4:**
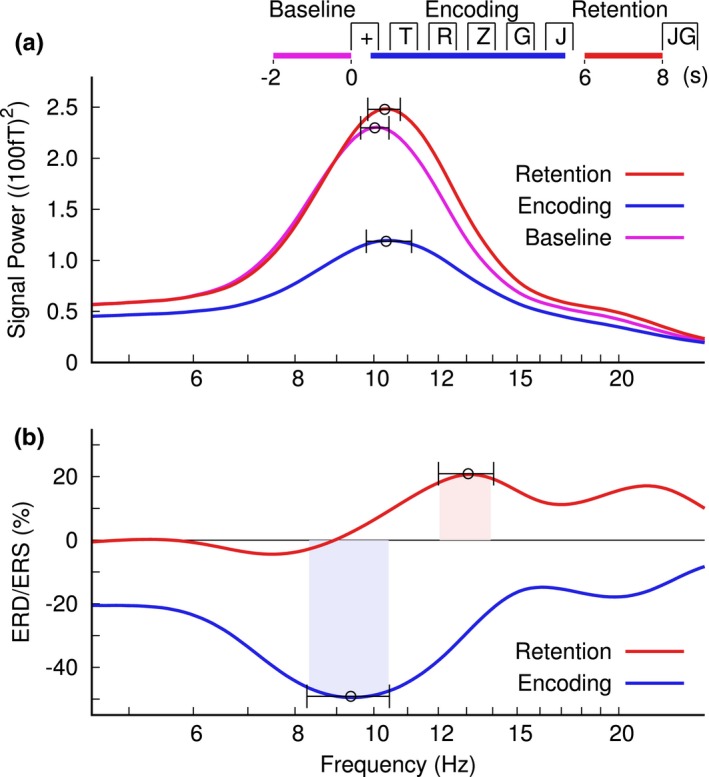
Alpha peak frequency in different time intervals of the working memory (WM) task at an example occipital sensor MRO21. (a) Alpha power in the baseline, encoding, and retention intervals. The error bars indicate the 95% confidence limits of the mean peak alpha frequency. (b) Alpha ERD and ERS in the encoding and retention intervals. Nonoverlapping confidence intervals indicate consistently higher frequency for the ERS peak in the retention interval compared to the ERD trough during encoding. The inset at the top indicates the time intervals for the spectrum analysis

Power spectra with the alpha peaks during baseline, encoding and retention intervals are illustrated in Figure 4a. The ANOVA for the peak alpha frequency revealed an effect of the time interval (*F*(2,48) = 6.27, *p* = 0.0038). The alpha frequency during encoding was higher than during baseline (*t*(149) = 5.32, *p* < 0.0001). Similarly, the alpha peak was at a higher frequency during retention compared to baseline (*t*(149) = 6.55, *p* < 0.0001). However, the alpha frequency was not different between encoding and retention intervals (*t*(149) = 0.08, *p* = 0.94, n.s.). Moreover, the ANOVA for the peak frequency in alpha power showed an effect of the sensor positions (*F*(5,120) = 14.2, *p* < 0.0001). The alpha frequency increased between frontal and central sensors (*t*(74) = 3.77, *p* < 0.0001) and between central and occipital sensors (*t*(74) = 4.78, *p* < 0.0001). There was also a “time interval” by “sensor group” interaction (*F*(10,240) = 3.93, *p* = 0.0001), because of effects of “time interval” in occipital sensors (*F*(2,48) = 9.98, *p* = 0.0002) and frontal sensors (*F*(2,48) = 3.88, *p* = 0.028) but not central (*F*(2,48) = 1.74, *p* = 0.19, n.s.).

The ERD/ERS spectra for encoding and retention are shown in Figure 4b. The ANOVA for the ERD/ERS peak frequency revealed an effect of the “time interval” (*F*(1,24) = 7.93, *p* = 0.0096). In mean across sensors, the peak alpha frequency was 9.5 Hz during encoding and 12.9 Hz during retention. An interaction of “time interval” and “sensor group” was significant (*F*(5,120) = 2.97, *p* = 0.015) because the alpha frequency was higher during retention compared to encoding right occipital (*t*(24) = 3.69, *p* = 0.0011) and left occipital* t*(24) = 2.49, *p* = 0.020) and as a tendency right frontal (*t*(24) = 1.78, *p* = 0.063), however not different at left frontal and central sensors.

### 3.5 Effect of memory load on alpha ERD/ERS

**Figure 5:**
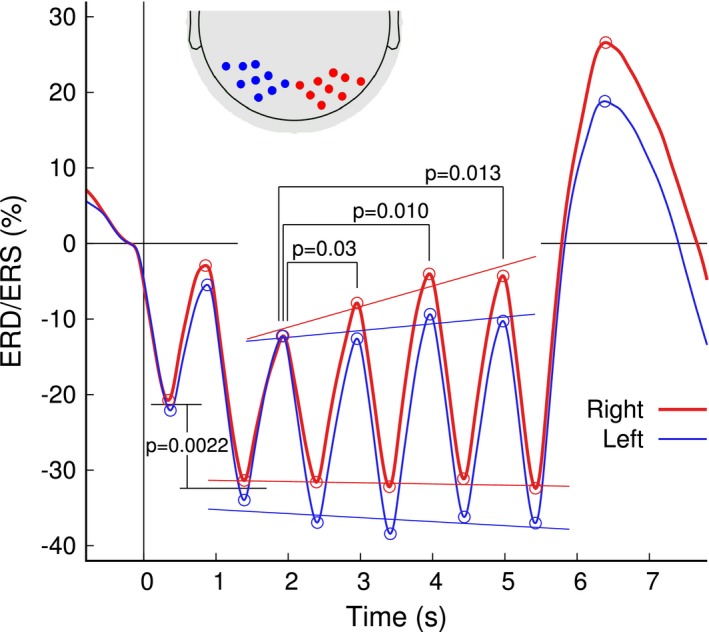
Time courses of alpha ERD/ERS in occipital sensors above the left and right visual cortices. The inset at the top depicts the selected clusters of MEG sensors. Amplitudes of peaks and troughs, indicated by open circles were analyzed across participants. Specifically, the peak amplitudes in the right hemisphere increased with increasing number of letters in the study list. In contrast, no significant change for the magnitude of ERD troughs was observed over the time course of presentation of the study list. The ERD induced by the list items was significantly larger than the ERD after the cue

Each visual stimulus elicited a brief period of alpha ERD followed by an immediate rebound. We tested whether the alpha ERD/ERS in sensors above the visual cortex depended on the stimulus sequence and thus could indicate involvement in encoding the increasing memory load. A repeated measures three‐way ANOVA for the peak ERD/ERS magnitudes with the factors “hemisphere” (left, right), “response type” (ERD trough, ERS peak), and “letter position” (1st to 4th), revealed an effect of “response type” (*F*(1,24) =6 6.2, *p* < 0.0001), which is trivial by definition of the peak types. More importantly, the ANOVA revealed a “letter position” by “response type” interaction (*F*(3,72) = 4.02, *p* = 0.011) and a “letter position” by “hemisphere” interaction (*F*(3,72) = 8.97, *p* < 0.0001). To unveil the causes of the interactions, separate two‐way ANOVAs with the factors “letter position” and “response types” were performed for the ERD/ERS magnitudes in the right and left hemisphere, respectively. The ANOVA for the right hemisphere revealed a “letter position” by “hemisphere” interaction (*F*(3,72) = 4.52, *p* = 0.0058) because the ERS peak magnitudes increased monotonically with increasing numbers of letters in the study list, while the magnitudes of ERD troughs remained in a steady level. The ANOVA for the left hemisphere showed only a tendency for a “letter position” by “hemisphere” interaction (*F*(3,72) = 2.51, *p* = 0.065). Pairwise comparisons found that subsequent peak amplitudes in the right hemisphere at 12 Hz were significantly larger than the first peak (positions 1–2: *t*(24) = 2.29, *p* = 0.031; positions 1–3: *t*(24) = 2.79, *p* = 0.010; positions 1–4: *t*(24) = 2.67, *p* = 0.013). The linear regression was significant for the right hemispheric peaks (*R* ^2 ^= 0.13, *F*(1,99)=14.6, *p* = 0.0002) but not for the troughs or the left hemispheric responses (Figure 5). Moreover, the analysis of the ERD peak magnitudes revealed that the cue stimulus elicited smaller ERD than the subsequent letter stimuli (*t*(24) = 3.15, *p* = 0.0022).

### 3.6 Alpha connectivity during the retention interval

**Figure 6:**
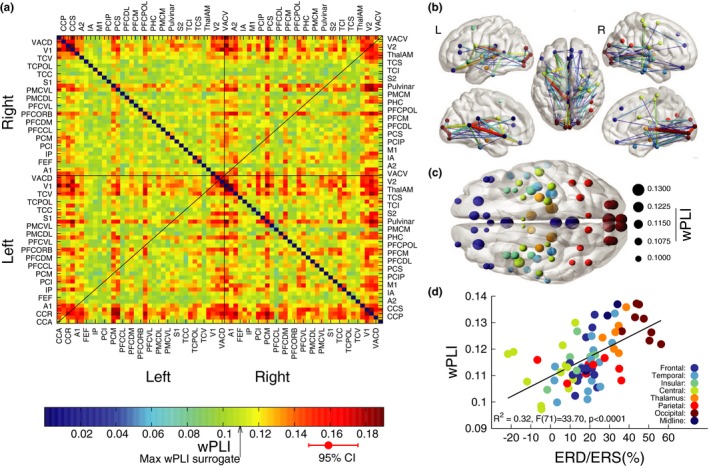
Connectivity during working memory (WM) retention. (a) Matrix of connectivity between 72 brain sources, measured the weighted phase‐lagging index (wPLI). An arrow at the color bar indicates the maximum wPLI observed in surrogate data. The red error bar shows an example of the 95% confidence interval of the mean across participants. Abbreviations of the brain sources: CCA, anterior cingulate cortex; CCP, posterior cingulate cortex; CCR, retrosplenial cingulate cortex; CCS, subgenual cingulate cortex; A1, primary auditory cortex; A2, secondary auditory cortex; FEF, frontal eye field; IA, anterior insula; IP, posterior insula; Ml, primary motor cortex; PCI, inferior parietal cortex; PCIP, cortex of the intraparietal sulcus; PCM, medial parietal cortex; PCS, superior parietal cortex; PFCCL, centrolateral prefrontal cortex; PFCDL, dorsolateral prefrontal cortex; PFCDM, dorsomedial prefrontal cortex; PFCM, medial prefrontal cortex; PFCORB, orbital prefrontal cortex; PFCPOL, polar prefrontal cortex; PFCVL, ventrolateral prefrontal cortex; PHC, parahippocampal cortex; PMCDL, dorsolateral premotor cortex; PMCM, medial (supplementary) premotor cortex; PMCVL, ventrolateral premotor cortex; S1, primary somatosensory cortex; S2, secondary somatosensory cortex; TCC, central temporal cortex; TCI, inferior temporal cortex; TCPOL, polar temporal cortex; TCS, superior temporal cortex; TCV, ventral temporal cortex; ThalAM, thalamus; V1, primary visual cortex; V2, secondary visual cortex; VACD, anterior visual cortex dorsal VACV, anterior visual cortex ventral. (b) Networks of 66 strongest connections. For the connections shown, the lower bound of wPLI was larger than the maximum obtained from the surrogate data. The networks are shown in a top view (middle panel) as well as lateral (top) and medial (bottom) sections in the left and right hemispheres. (c) Connectedness, defined as the mean across each row of the wPLI matrix, represented by the size of the circles. The color code relates to brain regions as explained in Panel D. (d) Correlation between connectedness and ERD/ERS for the 72 ROIs. Color code represents the different brain areas

The connectivity measure wPLI was computed for all 72 sources and resulted in a 72 × 72 matrix of group mean connectivity (Figure 6a). The elements along the main diagonal are zero by definition. Moreover, the main diagonal mirrors wPLI values in the upper and lower triangles of the matrix because of wPLI(x,y) = wPLI(y,x). A permutation test showed that overall connectivity was stronger during retention compared to the baseline interval (*p* < 0.0001). Visual inspection of the connectivity matrix revealed similar patterns within hemispheres (i.e., the quadrants along the main diagonal) and between homologue areas across hemispheres. Correlations between the quadrants of the connectivity matrix, excluding the midline connections, showed correlation between connectivity within left and right hemispheres (*R* ^2 ^= 0.467, *F*(1,560) = 1,012, *p* < 0.0001) as well as correlation between connectivity within the left hemisphere and interhemispheric connectivity (*R* ^2^  0.146, *F*(1,560) = 196, *p* < 0.0001), and between right hemispheric and interhemispheric connectivity (*R* ^2 ^= 0.114, *F*(1,560) = 148, *p* < 0.0001). Permutation tests showed that the overall connectivity within the left hemisphere was larger than the right hemisphere (*p* < 0.0001).

The group mean wPLI values were compared against the maximum value of 0.114 observed from randomized surrogate data. The network of the strongest connections is visualized in Figure 6b. Main effects (wPLI > 0.18) were seen in connections involving occipital, frontal, and mid‐brain sources. Local connectivity was prominent in the visual cortex with the strong (wPLI = 0.199) connections between the right primary visual cortex (V1) and the left secondary visual cortex (V2). Longer range connections were observed between visual cortices and the retrosplenial cingulate cortex as well as thalamic sources. However, connections between visual cortices and prefrontal cortices were numerically smaller (wPLI < 0.16). Prefrontal cortices were mostly involved in longer range connections involving the mid‐brain sources and temporal cortices whereas local connections were relatively weaker (wPLI < 0.15). An interesting feature of the network was that even though there was a strong connection between thalamic sources with anterior and posterior brain areas, direct connections between the two cortices were relatively weaker. This suggests that the thalamic structures might serve as a connection hub.

Visual inspection of the connectivity matrix in Figure 6a reveals that certain rows and columns of the matrix have larger values than others. This means that some individual sources are connected to many other sources while other sources are less involved in connectivity. Therefore, we calculated the mean across each row as a measure of connectedness. The strength of the connectedness is visualized for the 72 ROIs in Figure 6c. Large values were observed in occipital and frontal sources. Considering that alpha ERS in occipital and frontal areas was a prominent characteristic during the retention interval, one question was, whether such signal power increase could indicate a timing mechanism that helps to synchronize brain areas involved in holding information during the retention interval. The regression analysis between the ERD/ERS and connectedness across all 72 ROIs (Figure 6d) revealed that was positively correlated with ERS (*R* ^2 ^= 0.32, *F*(1,71) = 33.7, *p* < 0.0001).

### 3.7 Cross‐frequency coupling between alpha phase and gamma amplitude

**Figure 7:**
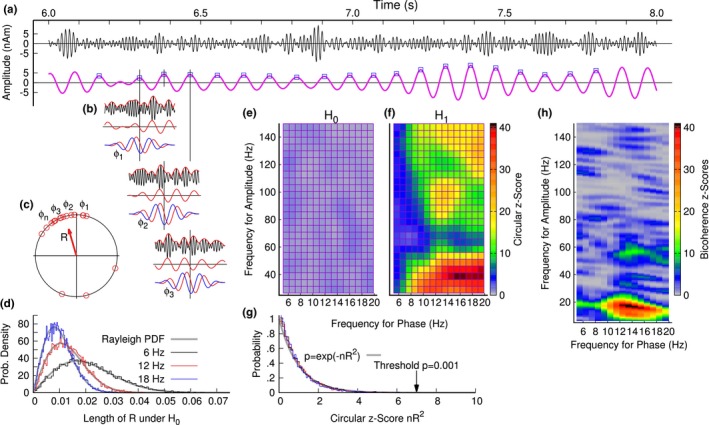
Cross‐frequency phase‐amplitude coupling. (a) Band‐pass filtered signals in the alpha (lower)and gamma (upper) frequency bands. (b) The gamma signal in short epochs, referenced to the alpha peak, was approximated by a complex wavelet at the alpha frequency. The phase of the wavelet served as a measure of phase relation between the gamma amplitude and the alpha peak. (c) Circular statistics for testing the phase relation between the gamma amplitude and the alpha phase. (d) Distribution of the vector length R, compared to the Rayleigh distribution (gray lines). The phase statistics depends on the number of trials. Alpha oscillations at 12 Hz contain a larger number of peaks than at 6 Hz. The larger number of samples results in smaller values for the phase statistics. (e) Comodulogram under the null hypothesis H_0_ obtained from surrogate data. (f) The comodulogram under H_1_ shows phase‐amplitude coupling between alpha and gamma frequencies. (g) The circular z‐scores are not dependent on the number of samples and allow of defining a global threshold for the comodulograms. (h) Bicoherence, calculated for the same data

The increase of alpha power during memory retention could indicate that alpha is involved in a timing mechanism of synchronizing gamma oscillations in different brain areas, which was tested with a cross‐frequency analysis. Figure 7 provides an overview about the flow of the analysis and the characteristics of the outcome measures. One trial of the band‐pass filtered data at low and high frequencies is shown in Figure 7a. The peaks of the alpha oscillations in time, marked by square symbols, served as phase reference for low‐frequency alpha signal. The amplitude of the high‐frequency gamma signal, time locked to the alpha peak, was approximated by a wavelet at the alpha frequency (Figure 7b). Across the alpha intervals and experimental trials, the phase of the wavelet approximation was uniformly distributed between −π and π. The phase statistics (Figure 7c) resulted in a measure of phase coherence, which was Rayleigh distributed under the null hypothesis (Figure 7d). The phase coherence was dependent on the number of trials, which was considered after transformation into circular z‐scores (Figure 7g). The comodulograms for one individual participant demonstrated large z‐scores for gamma frequencies around 40 Hz, between 80 Hz and 100 Hz, and above 120 Hz (Figure 7f). In contrast, the comodulogram resulting from surrogate data, simulating the null hypothesis, showed an even distribution of small z‐scores across the plane of gamma and alpha frequencies. For comparison, the comodulogram calculated for the same data with the bicoherence method (Figure 7h) showed large z‐scores for gamma frequencies below and above 40 Hz, which was the predominant maxima in Figure 7f. The bicoherence method, which assumes coherent high‐frequency oscillations and phase coherence between the sidebands of the amplitude‐modulation spectrum, showed no significant effects for higher gamma frequencies, in contrast to our time‐domain analysis. The differences between PAS measures obtained with the different algorithms allows for discussion about the underlying mechanisms.

### 3.8 Phase‐amplitude coupling (PAC) in the lower gamma band

**Figure 8:**
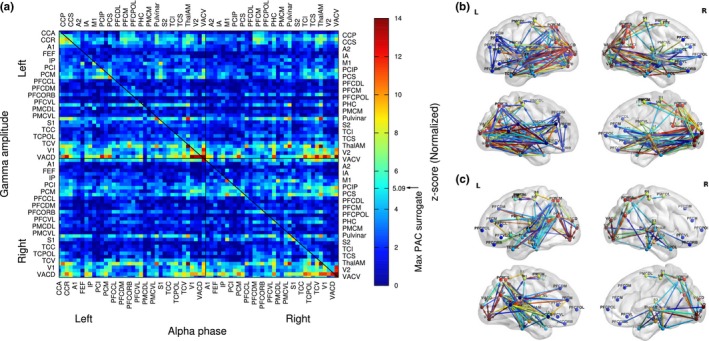
Alpha‐gamma phase‐amplitude coupling (PAC) during the retention interval. (a) Connectivity matrix of PAC for the 72 ROIs. Color coded are the normal z‐scores for the phase coherence between alpha and the gamma amplitude. The arrow at the color bar indicates the maximum PAC value which was observed from randomized surrogate data. In contrast to the wPLI matrix in Figure 6, the PAC matrix is not symmetric with respect to the main diagonal, that is, generally PAC(A;B)≠PAC(B;A). (b) Directed network PAC, indicating how the gamma amplitude in one source (arrow head) is coupled with the gamma phase in a second source (arrow tail). (c) Directed network of the reverse coupling of alpha phase (arrow head) with gamma amplitude (arrow tail)

The alpha‐gamma PAC at 13 Hz and 45 Hz, corresponding to the maximum in the comodulogram in Figure 7f, was calculated between all pairs of the 72 beamformer sources during the WM retention interval, resulting in a 72 × 72 connectivity matrix (Figure 8a). To illustrate the effect sizes, the full range of z‐scores between 0 and 14 is shown without truncating the data at a certain significance level. For comparison, the maximum z‐score of 5.09 was observed for the frequencies of interest across all 72 × 72 PAC values in randomized surrogate data and is indicated with an arrow at the color bar in Figure 8a.

The properties of the PAC matrix in Figure 8a were different from those of the wPLI matrix in Figure 6a. Specifically, the PAC matrix was not mirror‐symmetric to its main diagonal because in general it holds that PAC(a,b)≠PAC(b,a), while the wPLI is commutative. The main diagonal of this matrix represented local coupling between the alpha phase and gamma amplitude of the same brain signal. Matrix rows indicated how the gamma amplitudes of a certain signal were coupled to the alpha phase of the other signals. The columns indicated the coupling between the alpha phases of a signal with the gamma amplitude of all corresponding signals. The matric had been arranged, that the second and fourth quadrants corresponded to the intrahemispheric coupling whereas the first and third quadrants represent the interhemispheric PAC.

Directional networks have been visualized for the largest PAC values as a network of gamma‐alpha coupling (Figure 8b) and of alpha‐gamma coupling (Figure 8c). Figure 8b revealed predominant coupling between gamma amplitudes in the visual cortices and the alpha phase in distributed brain areas. Such schema of gamma‐alpha coupling relates to the rows of large PAC values for bilateral visual cortices in Figure 8a. The directed networks of alpha‐gamma coupling (Figure 8c) did not show a concentration around a certain source area and could be described as short‐range connections between distributed brain areas. Correspondingly, the PAC matrix did not show a pronounced columnar structure.

### 3.9 Phase‐amplitude coupling (PAC) in the higher gamma band

**Figure 9:**
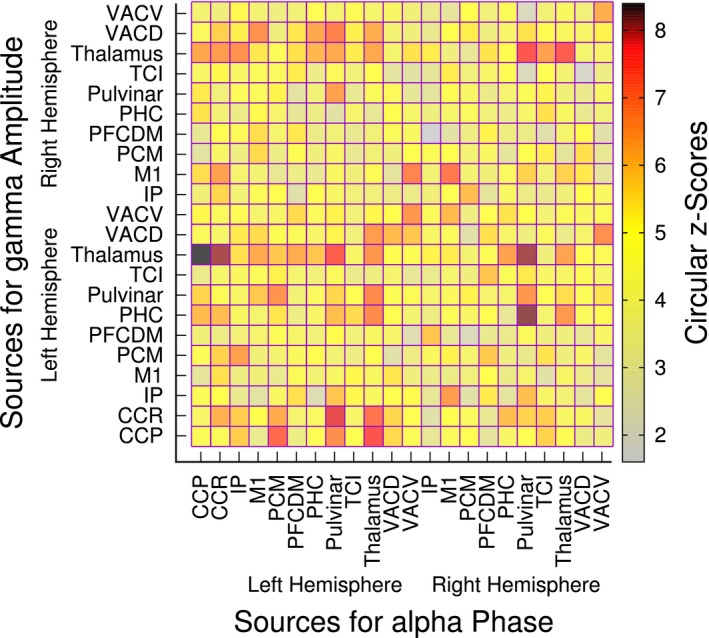
Group mean z‐scores for the phase‐amplitude coupling between 12–14 Hz alpha and 60–100 Hz gamma frequencies for selected sources showing strongest effect sizes

The group mean connectivity matrix for PAC between alpha phase and gamma activity in the 60 Hz to 100 Hz range is shown in Figure 9 for the sources with strongest effect sizes. PAC had been calculated for all 72 × 72 pairs of sources. The covariance of the connectivity matrix had been calculated and the sources with the largest values along the diagonal had been selected for Figure 9. Strongest PAC was observed for sources in the thalamus, posterior cingulate cortex, and visual cortices.

### 3.10 *Alpha‐gamma *versus*. gamma‐alpha* asymmetry

**Figure 10:**
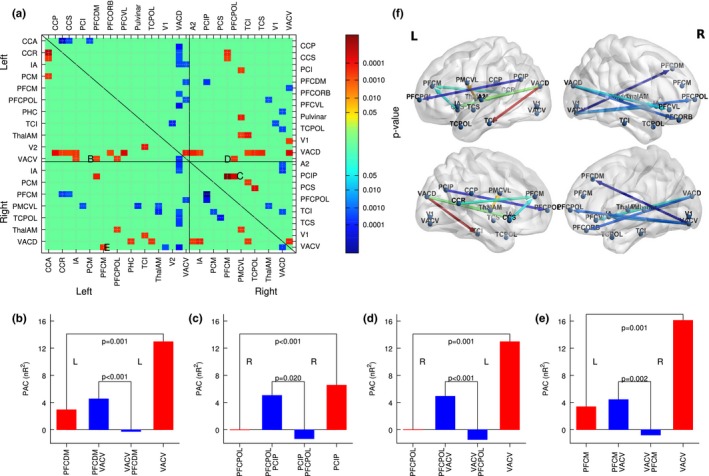
Asymmetry of PAC as an indicator for directionality of communication within brain networks. (a) The *t* tests, rejecting the hypothesis of PAC(A;B) = PAC(B;A). The labels indicate source pairs for which more details is provided in subpanels B‐E. (b) Asymmetric PAC between the anterior insula (IA) and the dorsal anterior visual cortex (VACD) within the left hemisphere. The red bars indicate the within same source PAC for the IA and VACD sources. The blue bars indicate PAC(IA; VACD) and PAC(IA; VACD), respectively, which were significantly different. (c) Asymmetric PAC between alpha phase of sources in the polar prefrontal cortex (PFCPOL) and intraparietal sulcus (PCIP) in the right hemisphere. (d) PAC between a pair of sources in the right PFCPOL and left ventral anterior visual cortex (VACV). (e) Asymmetric PAC between right PFCDM and left VACV. (f) Directional brain networks based of differences between local and distant asymmetry in their alpha‐gamma PAC. The networks reveal predominant coupling between anterior gamma amplitude (arrow tail) and frontotemporal alpha phase (arrow head). Temporal gamma amplitude was exclusively coupled to frontal alpha phase

The asymmetry between *PAC(a,b)* and *PAC(b,a)* was tested with two‐tailed *t* test for all pairs of nonidentical sources *(a,b)* and *(b,a)*. The outcome of the test is visualized with the matrix of FDR‐corrected p‐values in Figure 10a. Warm colors in this matrix indicate stronger gamma‐alpha coupling than alpha‐gamma coupling and cold colors vice versa.

Four examples for the asymmetry between alpha‐gamma and gamma‐alpha coupling are shown in detail in Figure 10b‐e. Each panel shows for a pair of sources (A,B), the within‐source PAC(A,A) and PAC(B,B) with red bars and the between‐sources PAC(A,B) and PAC(B,A) with blue bars. For the selected examples, the difference in between‐sources PAC was significant (*p* < 0.001 for all cases) and was accompanied by a significant difference in the within‐source PAC for the two sources. Also the same relationship of $\left(PAC\left(A,B\right)>PAC\left(B,A\right)\right)|\left(PAC\left(B,B\right)>PAC\left(A,A\right)\right)$. A regression analysis for all pairs of sources showed that the difference in between‐source PAC and the difference in within‐source PAC was negatively correlated (*R* ^2 ^= 0.30, *F*(1,2,554) = 1,091, *p* < 0.0001). In other words, the alpha‐gamma cross‐frequency coupling for a pair of sources was systematically determined by the strength of local within‐source coupling.

We further analyzed if the asymmetry in PAC could indicate the flow of information during memory retention. For this analysis, we considered local coupling as an indicator of neural processing and thus selected pairs of ROIs for which local coupling was significantly different as well as the inter‐ROI coupling. From 43 pairs of ROIs with difference in PAC (Figure 10a), 13 showed also differences in local coupling. Visualization of the resulting network in Figure 8f showed coupling of anterior gamma amplitude (arrow tail) and frontotemporal alpha phase (arrow head). Likewise, temporal gamma amplitude was exclusively coupled to frontal alpha phase.

## 4 discussion

Spectral analysis of the MEG during the processes of WM revealed a decrease in alpha power during memory encoding and a subsequent rebound above baseline level that correlated with the number of encoded items. The retention interval was characterized by increased alpha power in frontotemporal and occipital brain areas. Alpha phase synchronization identified occipital and frontotemporal brain areas as having the strongest overall connectivity to other brain areas. Importantly, during the retention interval, sensors with large alpha ERS also showed strong overall connectivity. Cross‐frequency coupling analysis between alpha phase and gamma amplitude during the retention interval revealed networks of short and long distances across the brain. The asymmetry property of PAS was introduced as a possible method for studying directionality in neural communication.

### 4.1 Reaction time (RT)

The behavioral performance measured with the RT showed the expected characteristic effects of primacy and recency and an increased RT when the probe was from items later in the study list. In case of the reverse probe order, RT was significantly longer than for the same order probe, while the RT dependency on the serial position was same. Thus, the reversed probe order required an additional process of mentally rotating the probe but did not affect memory performance itself. We are using the behavioral results here as confirmation that the participants performed the WM task. The relation between alpha oscillations and behavior will be reported elsewhere.

### 4.2 Alpha ERD during the encoding interval

Most prominent characteristic of alpha oscillations during the encoding interval was the sudden decrease in signal power compared to the baseline level, that is, an ERD, immediately following the onset of a visual stimulus. The ERD reached its deepest point at 260 ms after stimulus onset was stronger for the list items than the start cue, and most prominent in occipital sensors above visual cortices. Such sensory stimulation‐related alpha ERD has been reported for the visual system (Pfurtscheller, Neuper, Neuper, & Mohl, 1994) and other modalities like the auditory (Fujioka, Mourad, Mourad, & Trainor, 2011; Tiihonen et al., 1991) and somatosensory systems (Hari, Salmelin, Salmelin, Mäkelä, Salenius, & Helle, 1997; Stančák, 2006). Despite its strict temporal relation to the stimulus, the alpha ERD is not a primary sensory response. Characteristic for primary sensory responses is their spatial organization in the neocortex according to the spatial organization of the sensory organ, for example, retinotopic, tonotopic, and somatotopic organizations. However, a simultaneous recording of sensory evoked responses and alpha ERD showed that only the evoked response showed a somatotopic organization (Nierula, Hohlefeld, Hohlefeld, Curio, & Nikulin, 2013) suggesting the stimulus‐induced ERD does not reflect a stimulus‐specific primary response but is more likely supporting the conditioning of the sensory cortex. Alpha ERD has been linked to controlled access to information and attention control through inhibitory filtering (Klimesch, Fellinger, Fellinger, & Freunberger, 2011). The similar time courses of alpha ERD in the various sensory modalities suggest common alpha mechanism across sensory modalities. Generation and modulation of oscillatory activity have been studied widely on a microscopic level, which identified recurrent inhibitory thalamocortical networks as the origin of alpha oscillations (Steriade & Llinas, 1988). Those studies agreed that desynchronization indicates an active state of processing (Steriade, Gloor, Gloor, Llinás, Lopes da Silva, & Mesulam, 1990). Still, a wide gap exists between the understanding of oscillatory mechanism at small scale and the effects on mass activity as observed in EEG and MEG. One explanation of alpha desynchronization could be that local processing in primary sensory cortices results in multiple activities at specific phases, which in turn is reflected in the sum of more global mass activity as an effect of desynchronization (Pfurtscheller et al., 1994). While synchrony in neural networks serves as a mechanism of communication and binding, the opposite effect of desynchronization, also termed phase reset, seems necessary for dynamic reconfiguration of connectivity (Thatcher, North, North, & Biver, 2009). In line with those theoretical considerations, a model of ERD/ERS generation proposed a stereotypical pattern of an interval of ERD, which precedes and prepares for a subsequent active state of processing during an ERS interval (Lemm, Müller, Müller, & Curio, 2009). The observed stimulus‐related alpha decrease during the encoding interval corresponds to such concept of preparation for subsequent action.

Alpha ERD in response to the sensory input was also observed in frontotemporal sensors, although the magnitude was smaller than in occipital sensors. Such frontotemporal ERD could also be related to release from inhibition and preparation for specific processing. Moreover, prominent alpha ERD occurred in left central sensors above the sensorimotor cortex contralateral to the responding hand. Central ERD gradually increased over the encoding and maintenance intervals, and it became most strongly expressed after probe presentation and the actual response. Thus, the long‐lasting ERD increase may be explained with preparation for the movement required for a response, and this preparation seems to begin immediately with the beginning of the stimulus sequence as much as 10 s before actual movement execution. Alpha ERD during movement preparation has been reported previously and was interpreted as preparing for a motor task but does not reflect processing for the specific task itself (Deiber et al., 2012). The alpha ERD in the sensorimotor system may support the concept of a preparatory role of alpha ERD. Finally, alpha desynchronization had been observed even during anticipation of an event (Bastiaansen, Böcker, Böcker, Cluitmans, & Brunia, 1999; van Ede, Jensen, Jensen, & Maris, 2010), again emphasizing the role of preparation for further processing. In summary, we interpret the role of alpha ERD during the encoding interval as an active, stimulus‐induced state of preparation for subsequent information processing.

Alpha ERD in occipital sensors lasted shorter than the actual stimulus presentation and showed a steep rebound. If alpha ERD reflects a release from inhibition and thus facilitates sensory processing, there seem to be no need to return quickly into a state of inhibition. In contrast, the steep rebound could indicate a more active process. Increase in alpha power has been shown as an active inhibitory process of protecting an encoded stimulus from further interference (Bonnefond & Jensen, 2012). A novel finding of our study was that the magnitude of alpha rebound was related to the number of stimulus items within the encoded list and could support the hypothesis that the rebound indicates an active process. The peak amplitude of the alpha rebound increased with increasing memory load. In contrast, the troughs of alpha ERD maintained a constant magnitude.

An oscillatory model of WM proposed that cycles of gamma oscillation control the storage of items in memory and each cycle of low‐frequency theta or alpha oscillations scans the list of items for maintenance (Jensen & Lisman, 1998). The model accounted for RT data in the Sternberg experiment. Our finding of increased alpha rebound could indicate that an oscillatory alpha network is increasingly involved in memory encoding and maintenance with increasing load. This results is in general agreement with previous research that proposed a relationship between alpha power and encoding of new information (Doppelmayr, Klimesch, Klimesch, Stadler, Pöllhuber, & Heine, 2002; Klimesch et al., 1996). More specific, recent studies showed a relation between increased alpha power and memory load from intracranial recordings (Meltzer et al., 2008) and EEG (Hsieh, Ekstrom, Ekstrom, & Ranganath, 2011). While previous studies relied on spectral analysis, time‐frequency analysis in our study preserved the time course of alpha ERD/ERS and showed a clear dissociation of the effect of memory load between ERD and ERS intervals.

### 4.3 Different alpha frequencies

A further important finding was that the time course of alpha was correlated with the number of items within the study list only in the upper alpha band, centered around 12 Hz. This result corroborates a previous finding of load‐dependent phase locking of alpha that was maximal at 12 Hz (Schack, Klimesch, Klimesch, & Sauseng, 2005). Moreover, we showed that the center frequency of alpha ERS during WM maintenance was higher than the center frequency of the alpha ERD during encoding, while absolute power increase from baseline occurred similarly for both ERD and ERS intervals. A first dissociation between the lower alpha band (8–10 Hz) and upper alpha band (10–12 Hz) had been reported as topographically widespread activity for the first and focal activity for the latter in EEG recordings of a cognitive task (Klimesch, Pfurtscheller, Pfurtscheller, & Schimke, 1992) and a movement task (Pfurtscheller, Neuper, Neuper, & Krausz, 2000). The interpretation was that lower alpha serves general task demands while upper alpha is task specific. It has been speculated that activity in the upper alpha band predicts performance in memory and cognition. For example, a higher peak alpha frequency was correlated with larger memory capacity (Moran et al., 2010). However, whether absolute power in the upper alpha band or the amount of event‐related modulation is important seems strongly dependent on the specific task (Klimesch et al., 2006). Other authors labeled the power increase during memory maintenance as beta activity (Daume et al., 2017). The frequency band around 15 Hz, centered between the alpha and beta bands, has been also termed the beta_1_ band. Computational modeling showed that beta_1_ rhythms created cell assemblies through concatenation of cycles of beta and gamma oscillation, and such mechanism could underlay memory formation (Kopell, Whittington, Whittington, & Kramer, 2011). Our spectrum analysis showed ERS predominantly in the upper alpha and band informed us to focus on this frequency band for the subsequent analysis of connectivity and alpha‐gamma coupling.

### 4.4 Alpha ERS during the WM maintenance interval

An increase in alpha power during the WM maintenance interval had been reported previously (Jensen et al., 2002; Klimesch et al., 1999; Tuladhar et al., 2007; Van Dijk et al., 2010). Those reports inspired a range of new interpretations of the role of alpha oscillation beyond its control of inhibitory states. Alpha ERS has been shown to serve as an active inhibition for protecting the memory from distraction by further sensory input (Bonnefond & Jensen, 2012; Händel, Haarmeier, Haarmeier, & Jensen, 2011). Our data are in line with this interpretation. However, our study did not include a distraction paradigm, and thus we cannot test how much the alpha ERS contributed to inhibition of further input. In other studies, using a distracting stimulus did not increase alpha power; thus, the hypothesis of protecting the memory by inhibition of possible distraction was not supported (Poch et al., 2018; Schroeder et al., 2018) Another role of alpha ERS has been shown for the timing of WM‐related processing (Klimesch, Sauseng, Sauseng, & Hanslmayr, 2007). Specifically, nested theta/alpha and gamma oscillations have been proposed as a model for WM (Jensen & Lisman, 1998). Our data support those concepts, and we analyzed specifically the role of alpha oscillation for connectivity and cross‐spectral coupling with gamma oscillations.

### 4.5 Alpha ERS correlates with functional connectivity

Alpha phase synchronization had been considered to play a role in active neuronal processing by modulation of neuronal excitability that biases neuronal and behavioral responses to sensory stimuli (Palva & Palva, 2011). Such modulations might be important for inhibition of task‐irrelevant processes (Klimesch et al., 2007; Mazaheri & Jensen, 2010), executive control of behavioral responses (Klimesch et al., 2007), or even active task‐relevant processing (Von Stein & Sarnthein, 2000). If indeed alpha ERS relates to inhibitory processes, then it will imply that areas exhibiting ERS are under inhibition from other brain areas or are exerting inhibition on of other brain areas. Such a process will result in increased functional connectivity between the two areas. Our results showed that areas with strong alpha ERS also exhibited strong connectivity. Specifically, occipital and right frontotemporal brain areas, in which ERS was strongly expressed, were also functionally connected. Because occipital areas are responsible for processing visual sensory input, inhibition of processing of further input would be an effective way of retaining memory. However, it had been suggested that alpha ERS in frontotemporal brain areas will hardly be interpreted as inhibition of external input stimulus since frontal brain areas are not known to be involved in visual stimulus processing (Sauseng et al., 2005). However, frontal alpha ERS indicated that these areas were prevented from becoming involved in new activities if the memory task was ongoing. As such, alpha phase synchronization between frontotemporal and occipital brain areas could represent a functional network that helps to inhibit both internal and external distractions.

### 4.6 Cross‐frequency coupling

Cross‐frequency coupling is a relatively new approach to analyze functional connectivity. The methods are still under development and evaluation. Several recent papers showed that significant PAC measures could result from harmonic components of non‐sinusoidal alpha (Gerber et al., 2016; Lozano‐Soldevilla, Huurne, Huurne, & Oostenveld, 2016), sharp edge artifacts (Kramer et al., 2008), or phase‐to‐phase coupling (Hyafil, 2015) not related to the proposed timing relation between alpha and gamma oscillations. On the other hand, harmonics of alpha and beta might be functionally relevant (Kopell et al., 2011; Lozano‐Soldevilla, 2018). Thus, the results of cross‐frequency analysis have to be cautiously scrutinized before conclusions can be made. We found alpha‐gamma PAC predominantly in the lower gamma band around 45 Hz. The spectral representation of the amplitude modulated 45‐Hz gamma activity includes sidebands below and above 45 Hz. One caveat is that the first harmonic of alpha could be mistaken for the lower sideband and could result in apparent PAC. Our bicoherence analysis showed predominance of the lower sideband of 45‐Hz gamma instead of symmetric contribution from the upper and lower sidebands. Thus, we are aware that further analysis is required to confirm and validate the current results. The influences of harmonics of alpha had been demonstrated for PAC between frequency bands of the same signal. However, we showed alpha‐gamma PAC across distant sources. The effects of alpha harmonics are likely stronger for within‐source PAC. However, our PAC results were not specifically stronger for within‐source coupling than between‐sources. We take these findings as an argument against the hypothesis of nonlinear harmonics of alpha as the cause for PAC. The bicoherence did not show any effect at higher gamma frequencies. One explanation would be that bicoherence requires coherent oscillations and phase coherence between the frequency bands in the amplitude‐modulation spectrum. However, specifically high gamma activity may not consist of coherent oscillations but of short bursts or even single periods (Jones, 2016). Our time‐domain analysis was more sensitive to such type of gamma oscillations.

The effects of cross‐frequency coupling are small, and the observation is limited by the signal‐to‐noise ratio. Most current reports are about intracranial recordings with likely higher signal‐to‐noise ratio. Our findings of alpha‐gamma PAC in the MEG source domain contribute to the development and advancement of the cross‐frequency coupling approach.

### 4.7 Alpha‐gamma cross‐frequency coupling during memory retention

A recent review showed that currently, several authors shared the view that low‐frequency theta and alpha and high‐frequency gamma oscillations play distinct active roles during the retention phase of WM (Roux & Uhlhaas, 2014). However, not much is known about the interactions and joint function of alpha and gamma oscillations. Interactions between the two frequency bands could exist either locally within the same brain area or between the distant brain areas. Intracranial recordings from parietal brain areas revealed that local coupling between alpha phase and gamma magnitude was modulated by a behavioral task (Voytek et al., 2010). This was considered to reflect a mechanism for selection between communicating neuronal networks. Here, we demonstrated that alpha‐gamma PAC could be observed with MEG source analysis and we corroborated the findings that such PAC was largest in parietal and occipital regions. This findings of local PAC in occipital and parietal brain areas during the retention phase of WM is also in line with the opinion that alpha activity is associated with functional inhibition during retention of memory items, specifically by generating pulses of inhibition every 100 ms that alters ongoing activity thus limiting the processing of incoming visual information (Bonnefond & Jensen, 2015).

An interesting property of alpha‐gamma PAC between distant brain areas is that it is not reciprocal and thus provides information about the direction of flow of information. Previous evidence suggested that low‐frequency oscillations may drive cortical gamma rhythms (Canolty & Knight, 2010; Schroeder & Lakatos, 2009; Spaak, Bonnefond, Bonnefond, Maier, Leopold, & Jensen, 2012) implying information flow from alpha to gamma. A recent simulation and electrocorticography study supported the alternative that the gamma envelope may drive alpha oscillations (Jiang, Bahramisharif, Bahramisharif, Gerven, & Jensen, 2015), leaving the question of directionality open for debate. However, when considering cross‐spectral coupling as a mechanism of long‐range communication, it is more likely that brain areas exerting control will tend to modulate or serve as the timer for the activities multiple brain areas. On the other hand, it is unlikely that the activity of such controller area will be modulated or driven by other brain areas. Our results showed a hierarchy of dependency in which temporal gamma depended on frontal alpha phase and occipital gamma on temporal alpha. As such, frontal alpha phase had more dependencies compared to frontal gamma activity. This supports alpha phase as having more of a controller role.

One question about the underlying mechanism was whether alpha indeed controls directly distant gamma or alpha connectivity could result in synchrony between distant brain areas whereas PAC acts as a local mechanism. In case of direct control of distant gamma by alpha oscillation, a higher PAC between the two areas would be observed compared to the local PAC. Our results, however, showed a higher local PAC compared to PAC between the brain areas. Also, areas with PAC also showed increased synchrony. This supports a mechanism in which communication is established by phase synchrony and PAC represents local computation. This in line with the principle of communication in WM networks in which alpha establishes the long‐range connectivity and gamma is involved in local computation (Von Stein & Sarnthein, 2000). Moreover, long‐range PAC has been proposed as a mechanism through which different networks can communicate by altering the extracellular membrane potential in local cortical regions such that neurons will be more likely to fire during particular phases or phase network ensembles of low‐frequency oscillations (Canolty & Knight, 2010; Haider & McCormick, 2009; Klausberger et al., 2003). A recent study of alpha‐gamma PAC in WM found evidence for co‐occurrence of PAC and phase synchronization between left inferior temporal and left frontopolar cortices, suggesting that rather than establishing direct synchronization at higher frequencies, distant brain areas could rather indirectly coordinate high‐frequency activity by means of low‐frequency phase synchronization and local cross‐frequency coupling (Daume et al., 2017). Our analysis revealed similar findings of alpha phase coherence between left frontal and left temporal brain areas with left temporal gamma amplitude depending on the frontal alpha phase. We further found a similar interaction between frontal and occipital brain areas, which supports the co‐existence of phase synchronization and PAC, in facilitating long‐range communication. In summary, our PAC analysis provided support for an active role of alpha during WM maintenance through long‐range coordination of sensory processing in temporal regions and possible inhibition of distracting sensory input in occipital brain areas.

Alpha‐gamma PAC at higher gamma frequencies involved strongly the anterior thalamus and the pulvinar. Given the limited resolution of the MEG beamformer analysis, the source labeled pulvinar may include the lateral geniculate nucleus (LGN), receiving the visual input. The LGN communicates the visual input to the cortex, while the pulvinar region of the thalamus controls the information flow between cortical areas by receiving input from cortical regions and influences activity in other areas (Saalmann, Pinsk, Pinsk, Wang, Li, & Kastner, 2012; Theyel, Llano, Llano, & Sherman, 2010). It has been suggested that alpha oscillations are specifically involved in these thalamocortical communications (Klimesch, 2012). Therefore, our findings of alpha‐gamma PAC between cortical areas and the thalamus are encouraging.

## 5 conclusion

Analysis of spectro‐temporal and spatial properties of the MEG provided support that different types of alpha oscillations are involved in multiple neural processes underlying WM. Stimulus‐related power decrease in the lower alpha band indicated an active role in preparing sensory areas and frontotemporal memory areas for subsequent processes. Rebound oscillatory activity in the upper alpha band reflected inhibitory processes of protecting the memory from irrelevant sensory input. Moreover, the correlation between the magnitude of rebound and the number of item in the study list indicated involvement in the memory process. The most prominent increase of signal power in the upper alpha band, occurring during the retention interval in occipital and frontotemporal areas, was correlated with long‐range connectivity measures and suggests involvement in communication between distant brain areas based on synchronization and timing. Moreover, the cross‐frequency coupling between the phase of alpha oscillations and the amplitude of gamma oscillations support the role of upper alpha band activity for controlling neural timing. Asymmetry in the PAS provided directionality information and suggested that frontal and temporal alpha phase controlled occipital gamma amplitudes, which in turn were interpreted as indicating local processes of the WM task.

## CONFLICT OF INTEREST

The authors declare that the research was conducted in the absence of any commercial or financial relationships that could be construed as a potential conflict of interest.
